# Billing models for measuring nursing care in inpatient and outpatient settings: a scoping review

**DOI:** 10.1186/s12913-024-12116-3

**Published:** 2025-01-17

**Authors:** Marco Di Nitto, Francesca Napolitano, Michela Calzolari, Yari Longobucco, Vittorio Masotta, Francesco Zaghini, Rosaria Alvaro, Giancarlo Cicolini, Loreto Lancia, Duilio Fiorenzo Manara, Laura Rasero, Gennaro Rocco, Maurizio Zega, Beatrice Mazzoleni, Loredana Sasso, Annamaria Bagnasco

**Affiliations:** 1 Department of Health Sciences, University of Genoa, Via A. Pastore 1, Genoa, 16132 Italy; 2 Ospedale Policlinico San Martino, DEA, Largo Rosanna Benzi, 10, Genoa, 16132 Italy; 3 Department of Health Sciences, University of Florence, Viale Morgagni, 48, Florence, 50143 Italy; 4 Department of Clinical Medicine, Public Health, Life and Environmental Sciences, University of L’Aquila, Piazzale Salvatore Tommasi 1, Coppito, L’Aquila, 67100 Italy; 5 Department of Biomedicine and Prevention, University of Rome Tor Vergata, Via Montpellier, 1, Rome, 00133 Italy; 6 Department of innovative technologies in medicine & dentistry, “G. D’annunzio” University of Chieti-Pescara, Chieti, 66100 Italy; 7 Faculty of Medicine and Surgery, Vita-Salute San Raffaele University, Via Olgettina, 58, Milan, 20132 Italy; 8 Centre of Excellence for Nursing Scholarship, c/o Order of Nursing Professions (OPI) of Rome, Viale degli Ammiragli, 67 sc. B, Rome, 00146 Italy; 9 Degree Course in Nursing, Catholic University “Our Lady of Good Counsel”, Tirana, 1000 Albania; 10 Fatebenefratelli Isola Tiberina, A.Gemelli IRCCS University Hospital Foundation, Largo A. Gemelli, 8, Rome, 00168 Italy; 11 Humanitas University, Via Rita Levi Montalcini 4, Milan, Pieve Emanuele 20090 Italy; 12 Scientific Committee CERSI-FNOPI, Rome, 00184 Italy; 13 FNOPI Board Member, Rome, 00184 Italy

**Keywords:** Nursing costs, Nursing billing models, Nursing economic evaluation, Health care service

## Abstract

**Background:**

The rising cost of healthcare is a concerning issue for healthcare systems. The Diagnosis Related Group (DRG) system lacks direct consideration for costs related to nursing care. Therefore, to date there is no clear picture of billing models that consider also nursing activity when evaluating healthcare service costs or what factors related to nursing care affect the costs of healthcare services and would therefore need to be considered in billing models.

**Methods:**

A scoping review was conducted. We searched articles published from January 2000 to September 2023, in English, Italian and Spanish. We consulted MEDLINE (Pubmed), CINAHL (EBSCOhost), Web of Science (Clarivate), SCOPUS (Elsevier), ProQuest and Google Scholar, government sites and major international bodies (e.g., National Health Service NHS, World Health Organization WHO).

**Results:**

We included a total of 31 studies. The results highlighted 3 categories of factors related to nursing costs, which involve the organisation (e.g., nurse-to-patient ratio), nurses (e.g., skill mix, interventions), and patients (e.g., patient complexity, patient outcomes). All the billing models reported in the literature considered one or more of these categories to estimate nursing costs. The results also showed that appropriate management of organisational and nursing factors, such as staffing and skill mix, could improve healthcare service costs, nursing care or practice, and patient outcomes.

**Conclusions:**

This study sheds light on the multifaceted aspects of nursing care that should be considered in a specific, comprehensive, billing model. Additional testing of existing models to verify their effectiveness, as well as the organisation of a permanent committee (or Task Force) that develops a comprehensive billing model, are necessary to guide the formulation of new policies.

**Supplementary Information:**

The online version contains supplementary material available at 10.1186/s12913-024-12116-3.

## Background

Healthcare systems around the world are struggling to provide high-quality care while controlling expenses because of rising costs of the healthcare sector. This issue impacts both developed and developing countries and it is not specific to any nation or geographical area [1]. An ageing population and associated morbidities, the prevalence of chronic illnesses, and the rising demand for advanced medical services and technology are all contributing to the enormous and growing financial load that healthcare systems are facing [2]. To assess these expenses, sophisticated algorithms capable of considering all pertinent elements are needed, which is a challenging undertaking.

Current billing models analyse various elements such as the cost of medications, hospital stays, medical procedures, and more. Specifically, in Europe and the United States, a fixed reimbursement is provided through the Diagnosis-Related Group (DRG) system, a method for classifying hospital cases based on the patient's diagnosis and the resources required for treatment, which is then used to determine the payment. This system is used to standardise the services provided to patients based on medical diagnosis, medical services, comorbidities, resources used, etc. Consequently, each healthcare service receives a fixed reimbursement by governmental bodies based on the type and quantity of DRGs treated by that service. The idea is to provide a standardised method to reimburse healthcare providers according to the complexity and resource intensity of the care they deliver, aiming at controlling costs, promoting efficiency, and ensuring fair reimbursement across different patient cases. However, in this system, the classification of each DRG is primarily based on the primary diagnosis, followed by comorbidities, complications, age, gender, and length of stay (LOS), with the latter being a key factor that can significantly increase or decrease the cost of the DRG depending on its duration [3]. Previously, considering this system and the high healthcare costs, healthcare policies also aimed at reducing costs through penalising measures, for instance by reducing reimbursement for hospitals with excessively high rates of hospital readmissions within 30 days of discharge [4]. These policies raised questions about their effectiveness, as there are doubts regarding the real benefits on the direct work of healthcare professionals [5] and the resulting patient outcomes. Several questions remain open to improve actual billing models, including the use of non-punitive systems to change clinical and organisational behaviour and measuring how much nursing care affects hospital costs.

The DRG system does not directly account for nursing care, as it primarily focuses on classifying hospital cases based on diagnoses, procedures, comorbidities, and length of stay. This omission of nursing care costs contributes to variability in healthcare service expenses, as hospitals may incur different nursing costs for patients with varying care needs [6]. Previous studies have reported that the cost of nursing care can vary considerably even within the same DRG, if nursing care is not taken into account [7], and therefore billing models that also consider the complexity of nursing activities should be used [8]. Furthermore, financial models that consider the cost of nursing activities may lead to better nursing organisation models and better patient outcomes [9].

Nursing care involves physical, cognitive, emotional and organisational work [10], highlighting the complexity of nursing work and it has been calculated that nurses represent 20–30% of all hospital costs [8, 11]. Previous studies have identified several factors regarding nursing care that may affect hospital costs, such as nurse staffing levels [12], nursing skill mix [13] and nursing turnover [14]. However, organisational factors are not the only factors used for assessing the impact of nursing care on hospital costs and other potential performance indicators (which depend on the type of activity performed by the nurse) may affect these costs [15]. Several authors have examined possible models for measuring the burden of nursing care in terms of costs and patient outcomes [16, 17], regarding the role of nursing care. However, currently, there are no universally recognized billing models that accurately measure the impact and cost of nursing care, and it remains unclear which specific factors related to nursing practice should be considered in these models. Therefore, a major awareness of which ‘hidden factors’ of nursing care should be considered in the billing models (and not captured in current billing models), could potentially lead to an appropriate organisation of nursing activities, with a reduction of healthcare costs and improved patient outcomes.

The aim of this review was to examine the current literature to identify billing models that consider nursing activities and nursing interventions. By focusing on these overlooked aspects, we aim to contribute to a more comprehensive understanding of healthcare costs and inform strategies to ensure appropriate healthcare reimbursement models.

## Methods

### Study design

This was a scoping review. We followed the Joanna Briggs Institute (JBI) methodology for scoping reviews [18] and the Preferred Reporting Items for Systematic Review and Meta-Analysis Protocols (PRISMA-P) [19]. The final report was redacted according to the PRISMA extension for Scoping Reviews (PRISMA-ScR) [20]. The protocol of this review was registered on Open Science Framework (OSF, osf.io/kh5fv) [21].

### Review questions

This review was guided by three main research questions:

1. Which factors related to nursing practice inpatient or outpatient settings should be considered for billing models?
2. What billing models evaluated the healthcare cost considering the impact of nursing practice?
3. What are the patient outcomes related to inpatient or outpatient settings costs?

### Search strategy

The search strategy aimed at finding all relevant articles that described the cost of factors related to nursing activity and that should be considered in billing models in inpatient or outpatient settings. A preliminary search, aiming at finding all relevant index terms, was conducted on MEDLINE (PubMed). All the terms identified were used to create and refine a comprehensive search strategy (Additional file 1) by a trained researcher (MDN). Subsequently, the search strategy was confirmed and adapted to the other databases considered. Thus, MEDLINE (PubMed), CINAHL (EBSCOhost), Web of Science (Clarivate), SCOPUS (Elsevier), ProQuest and Google Scholar were searched from 2000 to September 2023. We also screened documents produced by governments or major international bodies (e.g., NHS, WHO and Independent Health and Aged Care Pricing Authority—IHACPA), along with the references of the articles that we included after full-text evaluation. Considering the languages spoken by at least one member of the research team, searches were limited to articles written in English, Italian and Spanish.

### Eligibility criteria

#### Concept

We considered all those studies that reported factors potentially related to nursing practice that could be considered to assess the nursing costs for inpatient and/or outpatient facilities. All studies that included a billing model or healthcare service reimbursement model in which nursing-related factors were also considered and with any type of objective (e.g., cost analysis, cost-effectiveness, cost utility, cost minimization, or cost benefit analyses) or outcome (direct nursing costs, indirect nursing costs) were considered. Studies that reported nursing-related factors but did not analyse or discuss them in terms of costs were excluded. For this scoping review, we considered as synonymous concepts of “billing model” also “reimbursement model” and “cost model”.

#### Context

We considered studies that evaluated factors of nursing practice associated with inpatient and outpatient costs. Thus, only these two settings were considered. Moreover, we included only studies conducted in high-income countries, as these counties are characterized by higher and more consistent healthcare expenditure, which supports standardized service organization and delivery. This approach reduces variability in the factors under investigation and ensures more robust comparisons, avoiding confounding effects associated with resource limitations and systemic heterogeneity often observed in low- and middle-income countries [22].

#### Type of sources

We included empirical, model-based studies, reviews, commentaries, and editorials reporting billing models for nursing practice or describing which nursing practice factors can be considered in billing models. We excluded conference abstracts without full manuscripts and case reports.

#### Study selection

**Fig. 1:**
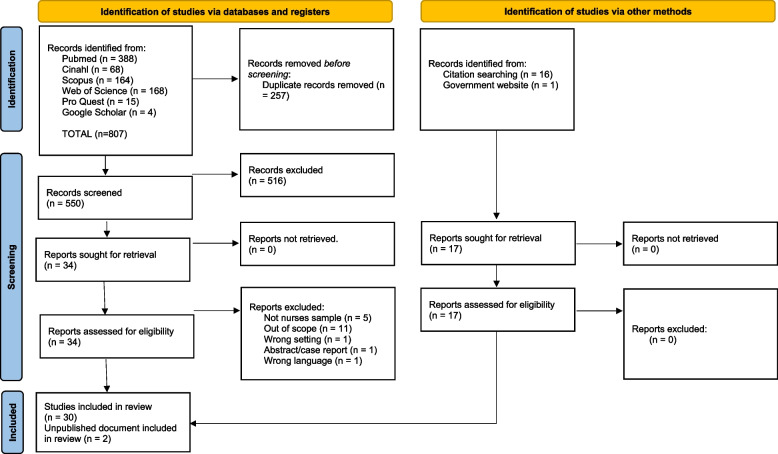
Study flow diagram

All articles collected through the search string were imported into Rayyan^©^ online software (Qatar Computing Research Institute, Doha, Qatar) and duplicates were removed. Three couples composed by two independent reviewers each screened all the titles and abstracts. The screening and selection phases were piloted on a random sample of 5 articles to ensure consistency with the eligibility criteria between the reviewers. Relevant papers included in the title-abstract screening phase were retrieved and the reviewers assessed the full texts in detail, considering the inclusion and exclusion criteria. At this stage, the reasons for exclusion were reported. The references of the included articles were searched for relevant articles and followed the same process. Disagreements at any stage of the selection process were resolved through discussion or consulting an independent reviewer. The study selection process is reported in the PRISMA flow diagram [23] (Fig. 1).

#### Data extraction

A summary table (data extraction tool) was developed by the research team to collect all relevant data. This table included all standard bibliographical data (e.g., authors, country, journal/source, design/type, context) and data related to the variables investigated (e.g., Nursing factors associated with healthcare service costs, Framework used, Main results, and Patient outcomes associated with the billing model). Specifically, we extracted data regarding frameworks described in the included studies that measured specific aspects of nursing care and which could have a potential impact on billing models. The data extraction tool was piloted on a random sample (n = 3) of included papers from the full list of records identified, to ensure that relevant information was being extracted. Data were separately extracted from the included papers by two reviewers.

#### Assessment of methodological quality

According to the JBI methodological manual [18], no evaluation of methodological quality of the included papers is required.

## Results

We included a total of 30 studies and two unpublished documents, of which 18 were from the USA [6, 17, 24–39], 4 from the UK [16, 40–42], 3 from Australia and New Zealand [43–45], 2 from Canada [46, 47], 2 from Belgium [7, 48], 1 from Japan [49], 1 from Ireland [50] and 1 from Norway [51]. The full list is shown in Table 1, including a narrative summary.

**Table 1:** Summary of general characteristics of included studies

**References**	**Country**	**Design/type**	**Context**	**Framework/instrument used**^**a**^	**Nursing factors related to healthcare service costs**			**Main results**		
					**Organisation**	**Nurses**	**Patients**	**Organisation**	**Nurses**	**Patients**
Knauf, 2006 [26]	US	Delphi study	Inpatient	NIW	-	Nursing Intensity	Patient complexity Patient outcomes	NIW can be associated with each DRG and obtain a comprehensive cost for the hospital	-	-
Heslop, L., & Plummer, V., 2012 [44]	Australia and New Zealand	Retrospective study	Inpatient	CNDMS NPR	Staffing	Nursing Hours	Patient dependency	The CNDMS underestimates the costs of nursing care as much as the NPR overestimates them	-	-
Titler, 2008 [32]	US	Retrospective study	Inpatient	-	Staffing	Nursing Interventions	Number of units resided on during hospitalization Percentage of time in intensive care unit	Higher RN/dip proportion (less hours dedicated to nursing care) was significantly associated with increased hospital costs	Performing fewer nursing interventions was significantly associated with increased hospital costs	Residing in two or more units was significantly associated with increased hospital costs
Welton, J. M., & Dismuke, 2008 [36]	US	Economic modelling study	Inpatient	Nursing Intensity Billing Model (based on NIW)	-	Nursing Hours Nursing Intensity	-	Adjusting DRGs for nursing intensity can improve the accuracy of hospital payment by adding a nursing component into inpatient billing systems	-	-
Welton, J. M., & Sermeus, W., 2010 [39]	US	Book Chapter	Inpatient	NMDS (based on NIW)	-	Nursing Hours Nursing Intensity	-	Nursing intensity data can be used for new measures of nursing remuneration and evidence-based staffing analysis	Nursing intensity can include nursing specific information in the claims and administrative databases and can be used to examine the clinical and cost outcomes of inpatient care	-
Welton, J. M., & Harris, K., 2007 [38]	US	Discussion paper	Inpatient	NMDS (based on NIW)	-	Nursing Hours Nursing Intensity	-	NMDS provides better models to predict staffing needs and analyse the cost of nursing care regardless of the medical diagnosis	-	-
Newbold D., 2008 [41]	UK	Retrospective secondary analysis	Inpatient	Production theory	Staffing	Staff mix	Patient outcomes	Nurse executives may use production theory to study the process and maintain quality or safety in the most economical way	-	-
Miyahira, A., 2015 [49]	Japan	Economic modelling study	Inpatient	NIS	-	Nurse tasks Nursing hours Nurse skills	-	It is possible to simulate the amount of nursing practices in the ward. E.g., analysing variable tasks to patients by Diagnosis Procedure Combination (Japanese DRG) enables the prediction of nursing tasks and nursing costs	-	-
Nuckols, 2017 [28]	US	Before-after study	Inpatient	Quality-cost framework	-	Nursing Hours Rounding practices	Patient outcomes	The fall prevention intervention that involved hourly rounding by RNs was effective in reducing fall risk and associated costs	-	Reduction of falls
Welton, J. M., & Harper, E. M., 2015 [37]	US	Preliminary work of Project 7	NP	-	-	Nursing Hours Nursing Intensity Nurse skills	Patient complexity	It is necessary to define components of nursing care value and appropriate methods to collect related data	-	-
Griffiths, P., 2021 [16]	UK	Simulation and Economic modelling study	Inpatient	Safer Nursing Care Tool	Staffing Organisational outcomes	Staff mix	Patient complexity Patient outcomes	The high baseline resilient staffing plan increased staffing costs compared to standard plan The low baseline flexible staffing plan reduced staff costs compared to standard plan	-	-
Ow, T. W., Ralton, L., & Tse, E., 2017 [43]	Australia and New Zeland	Retrospective audit	Outpatient	-	-	Nursing Intervention	Patient outcome	-	The activity (interventions) of the Nurse Coordinator resulted in economic saving	-
Botz, C. K., Sutherland, J., & Lawrenson, J., 2006 [46]	Canada	Retrospective study	Inpatient	-	Organisational outcomes	Nursing Hours Nursing workload	-	Common variations in case costing methodology can have a pervasive and material impact on derivative case-mix cost weights The precision of cost weights is inversely related to the level of averaging or use of standard costs	-	-
Welton, J. M., Fischer, M. H., DeGrace, S., & Zone-Smith, L., 2006 [6]	US	Retrospective secondary analysis	Inpatient	NID system	-	Nursing Intensity Nursing Hours	Patient complexity	Nursing care is an independent effect related to the cost of care and resources expended during hospitalization	-	-
Rudisill, P. T., 2014 [29]	US	Pilot study	Inpatient	HCIS	Organisational outcomes	Nursing Intensity Nursing outcomes (satisfaction, teamwork)	Patient complexity Patient outcomes	Proper discharge of lower-acuity patients, proper work allocation, and staffing-mix allocations resulting from workload rebalancing resulted in a reduction of costs	Improvement of nurse satisfaction Better teamwork communication	Improvements of patient outcomes
McGillis Hall, L., Doran, D., & Pink, G. H., 2004 [47]	Canada	Descriptive correlational study	Inpatient	-	Staffing	Nursing Hours Staff mix	Patient complexity Patient outcomes	A lower proportion of professional nursing staff (RNs/RPNs) was related to the use of more nursing hours The higher the proportion of unregulated staff on the unit, the greater the nursing hours costs	A lower proportion of professional nursing staff (RNs/RPNs) showed a higher number of medication errors and wound infections	More complex patients had positive influence on nursing hour utilization Older patients used more nursing hours Medical-surgical patients required more nursing care hours
Shamliyan, T. A., 2009 [30]	US	Meta-analysis	Inpatient	Nurse-sensitive patient outcomes	Staffing	Nursing sensitive outcomes	Patient complexity Patient outcomes	Increasing RN staffing was associated with a positive savings-cost ratio Hospital savings from reduced length of stay were less than the increased cost of RN staffing	-	Reduced length of stay
Welton, J. M., & Halloran, E. J., 2005 [37]	US	Retrospective secondary analysis	Inpatient	NMDS NDX	-	Nursing diagnoses	Patient complexity Patient outcomes	Considering NDX along with DRG can increase the explained variance for total hospitals charges	-	NDX are associated with patient outcomes (death, length of stay, ICU days and discharge to nursing home)
The Shelford Group, 2013 [42]	UK	Tool	Inpatient	-	Organisational outcomes	-	Patient complexity Patient outcomes	-	-	-
Andersen, M. H., 2016 [51]	Norway	Prospective comparative study	Inpatient	OPCq	-	Nursing Intensity Nursing Intervention	-	-	Predictor variables impacting Nursing Intensity were length of stay, ICU stay, and type of surgery	-
Chiang B., 2009 [24]	US	Methodological review	Inpatient	RCCs RVUs NIWs	-	- RVUs: Nursing Hours - NIWs: Nursing Intensity	- RCCs: acuity level - RVUs: acuity level - NIWs: Patient complexity Patient outcomes	NIWs used as the basis to estimate nursing care costs allow hospitals to have a better overall cost overview of their expenditures A more specific and accurate costing system, allows hospitals to make better pricing decisions and become more efficient in cost management	-	-
Dykes, P. C., 2013 [23]	US	Pilot study	Inpatient	- RVUs - CCC - Simple cost-to-time method (based on CCC)	-	- RVUs: Nursing Hours - CCC: Nursing Intervention Nursing Action Type (Assess; care; teach; manage) - Simple cost-to-time method: salary weight costs allocated for the inpatient nursing staff by the amount of time spent for each CCC action type	- RVUs: acuity level	RVUs method was less accurate and transparent than the simple cost-to-time method for costing nursing services	-	-
Lee, M., & Clancy, T. R., 2016 [27]	US	Retrospective study	Inpatient	GRASP System	-	Nursing Intervention Nursing Hours	Patient outcomes	-	Nursing interventions had greater explanatory power than severity of illness scores related to hospital care costs	-
Pirson, M., 2013 [7]	Belgium	Retrospective study	Inpatient	APR-DRG NMDS	-	Nursing Activity (Intensity)	Patient complexity Patient outcomes	The calculation of nursing cost by inpatient stay and by DRG should be based on nursing activity data and not on length of stay data, which does not reflect the variability of care	-	-
Sermeus, 2009 [48]	Belgium	Delphi study	Inpatient	APR-DRG NMDS	-	Nursing Weight Nursing workload	Patient Dependency	-	-	-
Welton, J. M., Zone-Smith, L., & Bandyopadhyay, D., 2009 [33]	US	Secondary data analysis	Inpatients	NID NPA	Staffing	Nursing Intensity Nursing Hours Nursing Characteristics (years of experience, academic preparation, etc.)	-	The calculation of direct nursing care hours based on NPA is a feasible and robust measure comparable to a nursing intensity estimate and better than methods that use mean unit or hospital estimates	-	-
Welton, J. M., Jenkins, P., & Perraillon, M. C., 2018 [34]	US	Editorial	Inpatient	TBAD NIW RVU NPA	TBAD: - key events in the care cycle (clinical and administrative components) - activity-based costing (dollar-per-minute capacity cost rate for each resource involved in the care cycle)	-	TBAD: Patient medical condition	-	-	-
Murphy, A., 2021 [50]	Ireland	Retrospective study	Inpatient	Framework for Safe Nurse Staffing and Skill Mix	-	Nurse sensitive adverse events	Patient outcomes Patient complexity (based on HPO)	Considering the economic impact of nurse sensitive outcomes must be taken into consideration when allocating resources	Each nurse sensitive adverse event increased the LOS beyond national average	-
Shever, L. L., 2008 [31]	US	Observational study	Inpatient	NIC	Number of Units resided on Time on ICU	Nursing Intervention Nursing Skill mix Nursing Hours	Patient characteristics	Integrating NIC in electronic health record allows the analysis of nursing care provided and its impact on direct cost and cost derived from adverse patient outcomes	High surveillance costs more than low surveillance delivery but may be associated with greater cost avoidance derived from adverse patient outcomes	-
Welton, J. M., Unruh, L., & Halloran, E. J., 2006 [6, 35]	US	Retrospective secondary analysis	Inpatient	-	Staffing	Nursing Intensity Nursing Skill mix Nursing Hourly Wage and Benefits	-	The distribution of patient-to-RN ratios, nursing intensity, and direct RN costs generally reflects different levels of care	Nursing resources care are variable depending on the hospital context	-
Griffiths, 2020 [16]	UK	Simulation and Economic modelling study	Inpatient	Safer Nursing Care Tool	Staffing	-	Patient outcomes	The Safer Nursing Care Tool is valid for estimating nurse staffing adequacy. Adequate nurse staffing improves cost per life saved and cost per patient-day	-	-
Independent Health and Aged Care Pricing Authority, 2024 [45]	Australia	Report	Inpatient	-	-	Nursing bucket (cost pools within a hospital)	-	-	-	-

*NIW* Nursing Intensity Weight, *CNDMS* Computer Nursing Dependency Management System, *NPR* Nurse-to-Patient ratio, *NMDS* Nursing Minimum Data Set, *EMR* Electronic Medical Record, *NIS* Nursing Information System, *QuadraMedCorp* minute-to-minute patient specific nursing workload data, *NID* Nursing Intensity Database, *HCIS* Evaluation of acuity systems, *NDX* Nursing Diagnosis, *OPCq* Oulu Patient Classification Instrument, *RVUs* Relative Value Units, *RCCs* Ratio of Cost-to-Charges, *CCC* Clinical Care Classification System, *GRASP* Systems International Companies, Fort Collins, CO, *APR-DRG* All Patient refined diagnosis related groups, *NPA* Nurse-patient assignment, *TBAD* time-based activity driven costing, *HPO* Health Pricing Office, *NIC* Nursing Intervention Classification

^a^That consider specific factors of nursing activity considered in a billing model

The majority of the included studies were retrospective or secondary analyses [7, 24, 25, 27, 33–35, 50, 51] or performed economic simulations or modelling [16, 36, 40, 50]. All the included articles except two [17, 43], considered the hospital inpatient setting.

In general, all the included studies recognised the importance of considering nursing costs and reported related factors associated with nursing care, and the great impact that nursing has on costs. The authors of the included studies used a variety of frameworks or instruments that considered specific factors of nursing activities in the billing models. Despite the differences between the frameworks of reference, the majority focused on estimating nursing intensity [6, 7, 16, 24–27, 33–36, 38, 39, 49, 51], or estimating the dependency or acuity of patients and related outcomes [7, 16, 24, 27, 29–31, 37, 44, 46, 50, 51]. Moreover, much attention was given to the possibility of using datasets that could collect and make available nursing-related data [6, 24, 25, 37–39, 48, 49, 51].

We then analysed the factors related to nursing activities that were considered for a billing model in inpatient or outpatient settings, and the outcomes derived from the consideration of nursing activities in the billing models. The respective results are reported below and grouped in the following categories: (I) organisation; (II) nurses; (III) patients. Overall, the studies considered similar factors and outcomes related to nursing activities, but they identified different dimensions related to the factors considered, which are reported in Table 2.

**Table 2:** Categories and sub-categories of nursing factors related to healthcare service costs

**Category**	**Sub-category**	**Nursing factors related to healthcare service costs**	**References**
Organisation	Staffing	Nurse-to-patient ratio	Heslop & Plummer, 2012 [44]; Titler et al., 2008 [32]; Newbold, 2008 [41]; Griffiths et al., 2020 [40]; Griffiths et al., 2021 [16]; McGillis et al., 2004 [47]; Shamliyan et al., 2009 [30]; Welton et al., 2006 [6]
		Average RN/patient ratio	Titler et al., 2008 [32]
		RN/dip proportion	Titler et al., 2008 [32]
		Patient assignment pattern	Welton et al., 2009 [33]
	Organisational characteristics/outcomes	Rounding practices	Nucklos et al., 2017 [28]
		Occupation	Griffiths et al., 2021 [16]; Shelford Group, 2013 [42]
		Ward Nursing Costs	Botz et al., 2006 [46]
		Number of sentinel events	Rudisill et al., 2014 [29]
		Number of near misses	Rudisill et al., 2014 [29]
Nurses	Nursing Interventions	Fluid Management	Titler et al., 2008 [32]; Dykes et al., 2013 [25]; Lee & Clancy, 2016 [27]
		Nutrition	Miyahira et al., 2015 [49]; Andersen et al., 2016 [51]; Dykes et al., 2013 [25]; Lee & Clancy, 2016 [27]
		Mobilisation	Miyahira et al., 2015 [49]; Lee & Clancy, 2016 [27]; Andersen et al., 2016 [51]; Dykes et al., 2013 [25]
		IV Therapy	Titler et al., 2008 [32]; Dykes et al., 2013 [25]; Lee & Clancy, 2016 [27]
		Pressure Ulcer Care	Titler et al., 2008 [32]; Dykes et al., 2013 [25]
		Planning/Care coordination	Titler et al., 2008 [32]; Lee & Clancy, 2016 [27]; Andersen et al., 2016 [51]; Dykes et al., 2013 [25]
		Hygiene and oral care	Titler et al., 2008 [32]; Miyahira et al., 2015 [49]; Lee & Clancy, 2016 [27]; Andersen et al., 2016 [51]
		Fall Prevention/Reduction	Titler et al., 2008 [32]; Nucklos et al., 2017 [28]; Lee & Clancy, 2016 [27]
		Bowel Management/elimination care	Titler et al., 2008 [32]; Lee & Clancy, 2016 [27]; Dykes et al., 2013 [25]
		Infection Protection	Titler et al., 2008 [32]; Dykes et al., 2013 [25]
		Medication Management	Titler et al., 2008 [31]; Rudisill et al., 2014 [29]; Miyahira et al., 2015 [49]; Lee & Clancy, 2016 [27]; Andersen et al., 2016 [51]; Dykes et al., 2013 [25]
		Education	Ow et al., 2017 [43]; Miyahira et al., 2015 [49]; Andersen et al., 2016 [51]; Dykes et al., 2013 [25]; Lee & Clancy, 2016 [27]
		Patient’s assessment	Miyahira et al., 2015 [49]; Lee & Clancy, 2016 [27]; Dykes et al., 2013 [25]
		Emotional support	Lee & Clancy, 2016 [27]; Andersen et al., 2016 [51]; Dykes et al., 2013 [25]
		Navigation	Ow et al., 2017 [43]
		Sleep and rest	Andersen et al., 2016 [51]
		Symptoms management/Pain control	Andersen et al., 2016 [51]; Dykes et al., 2013 [25]; Lee & Clancy, 2016 [27]
		Nursing Surveillance	Shever et al., 2008 [31]
	Nursing Intensity/workload	Nursing intensity described as Assessment; Teaching; Emotional; Medical; Physical	Knauf et al., 2006 [26]; Chiang, 2009 [24]
		Patients’ need for care and the nursing interventions needed to ensure good care	Andersen et al., 2016 [51]
		Advances in technology	Knauf et al., 2006 [26]
		Nursing care time—Direct nursing care hours per patient	Welton et al., 2009 [33]; Welton & Dismuke, 2008 [36]; Welton & Sermeus, 2010 [39]; Welton & Harris, 2007 [38]; Welton & Harper, 2015 [52]
		Mean hours of RN care per patient per shift	Welton et al., 2006 [6, 35]
		Ward-average per diems prorated by length of stay (in minutes) on each ward	Botz et al., 2006 [46]
		Minute-to-minute patient specific nursing workload	Botz et al., 2006 [46]
		Minutes of nursing care per stay	Pirson et al., 2013 [7]
	Nursing Hours	Nursing hours per patient per day (HPPD)	Heslop & Plummer, 2012 [44]
		Nursing care hours per patient	Welton & Dismuke, 2008 [36]; Welton et al., 2009 [33]
		Average nursing hours required for acuity level of patients	Chiang, 2009 [24]
		Hours per FTE (full-time equivalent) per year	Nuckols et al., 2017 [28]
		Hours of nursing labour	Nuckols et al., 2017 [28]
		RN hours of care in unit per year	Nuckols et al., 2017 [28]
		Case nursing hours (allocated to individual patients using workload measurement tools)	McGillis et al., 2004 [47]
		Hours benefit	McGillis et al., 2004 [47]
		Total of RN hours for a 1-h time period / total of patient hours for that same hour	Shever et al., 2008 [31]
	Staff mix/skill mix	Percentage of graduate RN’s	Newbold, 2008 [41]
		Skill mix between RN and assistant	Griffiths et al., 2021 [16]
		RN/registered practical nurse staff mix	McGillis et al., 2004 [47]
		All-RN staff mix	McGillis et al., 2004 [47]
		Proportion of regulated to unregulated staff	McGillis et al., 2004 [47]
		RN/registered practical nurse/unregulated staff mix	McGillis et al., 2004 [47]
		Proportion of RNs to all nursing direct caregivers for a specified period	Shever et al., 2008 [31]
		Percent hours of RN care per total staff care hours	Welton et al., 2006 [6, 35]
	Nursing sensitive adverse events	Urinary tract infection, pressure ulcers, hospital-acquired pneumonia, deep venous thrombosis, upper gastrointestinal bleeding, CNS complications, hospital-acquired sepsis, shock/cardiac arrest, wound infection, pulmonary failure, physiological/metabolic derangement	Murphy et al., 2021 [50]
	Nursing outcomes	Satisfaction	Rudisill et al., 2014 [29]
		Teamwork	Rudisill et al., 2014 [29]
Patient	Patient outcomes	Safety and security	Knauf et al., 2006 [26]
		Length of stay	Rudisill et al., 2014 [29]; Griffiths et al., 2021 [16]; Shelford Group, 2013 [42]; Shamliyan et al., 2009 [30]; Welton & Halloran, 2005 [37]; Pirson et al., 2013 [7]; Murphy et al., 2021 [50]
		Pain management	Knauf et al., 2006 [26]
		Skin integrity	Knauf et al., 2006 [26]; Titler et al., 2008 [32]; Griffiths et al., 2021 [16]; Shelford Group, 2013 [42]; Rudisill et al., 2014 [29]
		Restrain-free care	Knauf et al., 2006 [26]
		Smoking cessation	Knauf et al., 2006 [26]
		Discharge management	Knauf et al., 2006 [26]; Welton & Halloran, 2005 [37]
		Involvement of families	Knauf et al., 2006 [26]
		Medication administration	Knauf et al., 2006 [26]; Griffiths et al., 2021 [16]; Shelford Group, 2013 [42]; Rudisill et al., 2014 [29]; McGillis et al., 2004 [47]
		Infection control	Knauf et al., 2006 [26]; Griffiths et al., 2021 [16]; Shelford Group, 2013 [42]; McGillis et al., 2004 [47]
		Isolation	Knauf et al., 2006 [26]
		Bed days	Griffiths et al., 2021 [16]; Shelford Group, 2013 [42]
		Severity of illness	Lee & Clancy, 2016 [27]
		Mortality	Newbold, 2008 [41]; Welton & Halloran, 2005 [37]
		Survival	Newbold, 2008 [41]
		Satisfaction	Knauf et al., 2006 [26]; Griffiths et al., 2021 [16]; Shelford Group, 2013 [42]; Ow et al., 2017 [43]; Rudisill et al., 2014 [29]
		Falls	Titler et al., 2008 [32]; Nucklos et al., 2017 [28]; Griffiths et al., 2021 [16]; Shelford Group, 2013 [42]; Rudisill et al., 2014 [29]; McGillis et al., 2004 [47]
		Accesses/readmissions	Griffiths et al., 2021 [16]; Shelford Group, 2013 [42]; Ow et al., 2017 [43]; Rudisill et al., 2014 [29]
		cost per patient-day	Griffiths et al., 2020 [16]
		cost per life saved	Griffiths et al., 2020 [16]
		Number of procedures	Ow et al., 2017 [43]
	Patient complexity/dependency	Mobility	Heslop & Plummer, 2012 [44]; Miyahira et al., 2015 [49]
		Nutrition	Heslop & Plummer, 2012 [44]; Griffiths et al., 2021 [16]; Shelford Group, 2013 [42];
		Hygiene	Heslop & Plummer, 2012 [44]
		Thought processes	Heslop & Plummer, 2012 [44]
		Number Needed to Treat/Number Needed to Harm	Griffiths et al., 2021 [16]
		Staff cost / life	Griffiths et al., 2021 [16]
		Net cost / life	Griffiths et al., 2021 [16]
		Severity of illness	Pirson et al., 2013 [7]; Shever et al., 2008 [31]
		Comorbidities	Shever et al., 2008 [31]

### I. Organisation

With regard to the category “organisation”, most of the included studies highlighted the importance of nurse staffing (nurse-to-patient ratios) on costs [16, 30, 32, 33, 35, 41, 44, 47]. Organisational factors that could be related to nursing (e.g., rounding practices, ward nursing costs, number of near misses, number of units resided on ICU) were also identified [16, 28, 29, 31, 42, 46].

The outcomes derived from the consideration of nursing in the billing models included a better allocation of nursing resources in terms of staffing, and a reduction in costs of nursing care [16, 28–30, 32, 38, 40, 41, 43, 47]. Moreover, an improvement in accuracy of hospital payment prediction and allocations was also highlighted [6, 17, 26, 36–39, 46, 49].

### II. Nurses

The factors identified in this category were nursing hours [6, 24, 25, 28, 31, 33, 36, 38, 39, 44, 46, 47, 49, 50, 52], nursing intensity [6, 9, 24, 26, 29, 33, 35, 36, 38, 39, 51, 52], nursing interventions or practices [25, 28, 31, 32, 43, 49–51], staff mix and nursing skills [16, 31, 35, 41, 47, 49, 52], workload [37, 46, 49], nursing weight [48], nurse sensitive adverse events [50], nurse characteristics [33], satisfaction and teamwork [29] and nursing bucket (cost pools within a hospital, i.e., nursing salaries and wages) [45].

The main results identified several predictors of clinical and cost outcomes, such as nursing interventions [27, 31, 32], nursing intensity [33, 39], staff mix [47], nurse sensitive adverse events [50], and nurses’ satisfaction [29].

### III. Patients

The identified nursing factors related to patients, included mainly patient complexity and patient outcomes [6, 7, 16, 24–30, 34, 37, 41–43, 47, 50, 52]. Specifically, patient outcomes were related to all those outcomes deemed important for patients that may have an impact on nursing activity or that could be caused by the nursing activity, thus impacting on billing models. Other factors included patient dependency [44, 48], number of units resided on during hospitalization and time in ICU [32], cost per patient-day, and cost per life saved [40], and patient characteristics [31].

The results of included studies mainly concerned improvement in patient outcomes and consequently on costs [28–32, 37, 47].

#### Billing models that evaluate the impact of nursing practice

Several models were identified in this review such as Nurse Intensity Weight (NIW) [24, 26, 34, 36], Computer Nursing Dependency Management System (CNDMS) [44], Nursing Intensity Billing Model (NIBM) [36], Nursing Minimum Data Set (NMDS) [7, 37–39, 48], Nursing Information System (NIS) [49], The Safer Nursing Care Tool (SNCT) [16, 42] and the Australian National Hospital Cost Data Collection (NHCDC) [45]. Each model has various characteristics, which are different or similar to the other models.

The Nurse Intensity Weight (NIW) was developed by a group of nurses to evaluate the costs of nursing care upon the patient’s discharge [24, 26]. The NIWs are based on the estimated costs of nursing care workload related to a DRG. In fact, the NIW Patient Classification Criteria associate a nursing score for each DRG. The nursing score is based on six dimensions of nursing care: Assessment, Planning, Teaching (patient and/or family), Emotional support (patient and/or family), Medical needs, and Physical needs [26]. Each dimension have a score based on a Likert scale from 1 (minimum) to 5 (maximum/more complex) [24, 26, 36]. The strength of the NIW is the association between nursing care costs and DRG, whereas the main disadvantage is that it lacks the variability and personalisation of nursing care [36].

The Computer Nursing Dependency Management System (CNDMS) is a cost modelling for nursing care based on patient dependency and the time required to care for 40 categorised specific types of patients, such as medical, surgical, psychiatric, or short stay patients. [44], The level of patient dependency is scored though five categories, which reflect all aspects of nursing care. In addition, nurse documentation, patient enquiries, simple medications, tutoring, relationship with patients, and doctors’ rounds are also included in this cost modelling [44].

The Nursing Intensity Billing Model (NIBM) is based on the NIW model. This model differs from the NIW model in that it allocates all direct nursing costs entailed in routine or intensive care costs, applying fixed costs (023X) for each day of stay [36]. This model enables to capture the variable of nursing time and charge.

The Nursing Minimum Data Set (NMDS) is an economic model based on Nursing Diagnoses, Nursing Interventions (NIC), Nursing Outcomes (NOC), and Nursing Intensity [37–39, 48]. Based on the NIC and NOC classification [48], this economic system is recognised internationally. Moreover, through the NMDS it is possible to evaluate nursing care costs in hospital similarly to DRG costs [38, 39]. This system is mainly used in Belgian hospitals [48].

The Nursing information system (NIS), reported only by one study [49], categorizes nursing care into three tasks, based on job indicators, which the authors did not specify. Miyahira et al. [49] simulated the cost of nursing practices by combining the Japanese DRGs with nursing tasks and costs.

The Safer Nursing Care Tool (SNCT) is an economic model used in English National Health Service Hospitals [16, 40]. This model simulates the staffing levels presented in each unit and shift. The staffing level is measured with a patient classification system based on patient complexity and outcomes [42]. With the SNCT it is possible to evaluate the cost per life saved using the effects of staffing on length of stay and the derived risk of death [16, 40].

The Australian National Hospital Cost Data Collection (NHCDC) is an annual initiative that gathers cost data from each state, territory, and private hospital in Australia. This collection links patient-level activity with the corresponding hospital costs and serves as the primary dataset for determining the national efficient price for funding public hospital services [53]. Hospitals submit cost data to the NHCDC using cost buckets, which represent specific cost categories within hospital activities. Nursing costs are identified through the nursing cost bucket, which encompasses nurses' wages and salaries [45].

## Discussion

The aim of this scoping review was to summarise available evidence regarding the billing models that take into account nursing activities, and the factors related to nursing costs.

The assessment of nursing care costs within healthcare systems remains a complex challenge due to the absence of universally applicable models. Some models for quantifying the nursing activities were reported (e.g., Nursing Intensity Weight; Nursing Minimum Data Set). Currently, only one model (the NHCDC implemented in Australia) [45] consistently accounts for nursing costs by considering nursing wages and salaries. Notably, this model is reviewed annually with the involvement of multiple stakeholders, aiming to enhance cost determination and improve reimbursement efficiency [54]. Regarding nursing activities, to the best of our knowledge, only one study [52] reported the involvement of an expert panel for considering factors related to nursing activities (e.g., nursing intensity, Nursing Relative Value Unit) that should be integrated in a novel reimbursement model. Further testing of current models to ascertain their efficacy, along with establishment of a standing committee (or taskforce) that develops a comprehensive billing model is necessary to guide policy makers. However, we realise that integrating the multifaceted aspects of nursing care, system organisation, and nursing dynamics make it very challenging to devise comprehensive models.

This study has shed light on the multifaceted aspects of nursing care related to billing models. The studies included in the present review were divided into three primary categories of influencing factors: organisational, nursing staff-related, and patient-oriented factors. Within these categories, factors like skill mix, nurse staffing levels, patient complexity, and specific patient outcomes consistently emerged as pivotal determinants impacting on the quality and cost-effectiveness of nursing care.

These aspects have been extensively analysed in the literature and results of interest to health systems have been reported. Skill mix and appropriate nursing staffing levels are associated with better patient outcomes [55] and a recent systematic review of 27 studies [56] confirmed that adequate nurse staffing levels have a beneficial effect on preventing patient mortality. Likewise, skill mix is also a factor that needs to be considered in a reimbursement system for healthcare facilities, as this factor can also be associated with different patient outcomes, such as pneumonia, sepsis, urinary tract infection, and length of stay [57], consequently impacting on healthcare costs. Moreover, included studies showed that patient complexity (identified in the studies by mainly considering the dimensions of mobility, nutrition, elimination, and hygiene) was also considered as a factor that can generate a greater nursing care burden and consequently a major costs of nursing care. Patient complexity implies greater utilization of health care services, resulting in higher costs, both in terms of materials, facilities, and the activities of health care professionals. In 2022, the cost of health care per capita in the countries of the Organisation for Economic Co-operation and Development (OECD) averaged nearly USD 5000, with a peak in the United States with the equivalent of USD 12555 for every citizen [58]. This cost may be partly due to the utilization of health care services by more complex patients. In the United States (the country that spends the most in terms of USD per capita), the number of avoidable diabetes admissions is higher than that of the OECD average [59]. The age of the population also plays a key role in increasing patient complexity, as the incidence of chronic diseases increases with older age and consequently a greater use of health care services due to increased complexity of the health conditions [60]. Therefore, countries reporting a higher average age (e.g. Japan, Germany, and Italy) could expect lower spending with a younger population, on average [58].

All these aspects that emerged from the present scoping review, are part of a complex picture that needs to be carefully considered. Within DRG systems, the potential hidden nursing care cost variability has significant ramifications for healthcare expenditure. A model that includes nursing care costs, based on nursing activities provided, could potentially guide resource allocation policies more effectively. In countries where health care is primarily financed through government-funding schemes (e.g., Canada, Italy, and UK), understanding these costs could empower organisations to identify and invest in areas pivotal to reduce expenses linked to adverse patient events, subsequently mitigating increased healthcare utilization.

Billing models that consider the weight and variability of nursing care for the same medical condition could pave the way for judicious resource allocation strategies. The rethinking the DRG model, or the consideration of a counterpart model that considers nursing-related factors affecting health care costs is necessary to meet current health care challenges.

### Limitations

Some limitations should be considered. First, despite efforts to exhaustively search for relevant studies, it is possible that some studies may have been missed, leading to the exclusion of other potential factors related to the cost of nursing in this article. In particular, there might be more grey literature on this topic. Another limitation might be that we considered only three languages in this study, so this inclusion criterion could have excluded articles with potential useful information.

## Conclusions

Integrating and adapting costing models that effectively and reliably document both the cost of medical interventions and nursing activities can lead to adequate reimbursements and more investments for healthcare services, avoiding a generalized and inadequate cut of resources. Considering the recent changes that are taking place in the health care sector (continuous cuts in health care funding, staff shortages, and an increase in chronic patient illnesses) nursing care cannot be considered second-rate in terms of cost, and adequate investment in implementing cost models for nursing care could improve processes and reduce care costs.

## Supplementary Information

Supplementary Material 1.

## Data Availability

All the data of this articles have been reported within the main manuscript or in supplementary materials. The protocol of this scoping is available at Open Science Framework (osf.io/kh5fv).
