# Supplementary material for: Billing models for measuring nursing care in inpatient and outpatient settings: a scoping review
Source: BMC Health Serv Res. 2025 Jan 17;25:95. doi: 10.1186/s12913-024-12116-3 (PMC11740332; doi:10.1186/s12913-024-12116-3)
Supplement: Supplementary file 1 — Supplementary Material 1. [file 12913_2024_12116_MOESM1_ESM.docx]

**Billing models for measuring nursing care in inpatient and outpatient settings: a Scoping Review**

**Additional file 1**. Search strategy used for each electronic database.

| **Search string** | **Records retrieved** | **Database** | **Date** |
| --- | --- | --- | --- |
| (("hospitals"[MeSH Terms] OR "outpatients"[MeSH Terms] OR hospital OR clinic OR "medical institution" OR "institutional care" OR "residential care" OR ambulatory OR outpatient OR inpatient) AND ("cost accounting mode*" OR "billing model*" OR "reimbursement model*" OR "cost model*" OR "charge model*" OR "charges model*" OR "cost effective model*" OR "economic model*")) AND ("nurses"[MeSH Terms] OR nurse OR nurses OR nursing OR nurs*) | 388 | MEDLINE (PubMed) | 7-9-2023 |
| ( nurse OR nurses OR nursing OR nurs* ) AND ( ("cost accounting mode*" OR "billing model*" OR "reimbursement model*" OR "cost model*" OR "charge model*" OR "charges model*" OR "cost effective model*" OR "economic model*" ) AND ( hospital OR clinic OR "medical institution" OR "institutional care" OR "residential care" OR ambulatory OR outpatient OR inpatient ) | 68 | CINAHL (EBSCOhost) | 7-9-2023 |
| Article Title, Abstract, Keywords (nurse OR nurses OR nursing OR nurs*) AND Article Title, Abstract, Keywords ("cost accounting mode*" OR "billing model*" OR "reimbursement model*" OR "cost model*" OR "charge model*" OR "charges model*" OR "cost effective model*" OR "economic model*") AND Article Title, Abstract, Keywords (hospital OR clinic OR "medical institution" OR "institutional care" OR "residential care" OR ambulatory OR outpatient OR inpatient) | 164 | SCOPUS (Elsevier) | 7-9-2023 |
| ((ALL=(nurse OR nurses OR nursing OR nurs*)) AND ALL=("cost accounting mode*" OR "billing model*" OR "reimbursement model*" OR "cost model*" OR "charge model*" OR "charges model*" OR "cost effective model*" OR "economic model*")) AND ALL=(hospital OR clinic OR "medical institution" OR "institutional care" OR "residential care" OR ambulatory OR outpatient OR inpatient) | 168 | Web of Science (Clarivate) | 7-9-2023 |
| (abstract(nurse OR nurses OR nursing OR nurs* ) OR title(nurse OR nurses OR nursing OR nurs* )) AND (abstract(("cost accounting mode*" OR "billing model*" OR "reimbursement model*" OR "cost model*" OR "charge model*" OR "charges model*" OR "cost effective model*" OR "economic model*") OR title(("cost accounting mode*" OR "billing model*" OR "reimbursement model*" OR "cost model*" OR "charge model*" OR "charges model*" OR "cost effective model*" OR "economic model*")) AND (abstract(hospital OR clinic OR "medical institution" OR "institutional care" OR "residential care" OR ambulatory OR outpatient OR inpatient) OR title(hospital OR clinic OR "medical institution" OR "institutional care" OR "residential care" OR ambulatory OR outpatient OR inpatient)) | 15 | Pro Quest | 7-9-2023 |
| allintitle: nurse "cost accounting model" OR "billing model" OR "reimbursement model" OR "cost model" OR "charge model" OR "charges model" OR "cost effective model" OR "economic model" "cost model" | 4 | Google Scholar | 7-9-2023 |
